# Implementing HIV/AIDS Education: Impact of Teachers' Training on HIV/AIDS Education in Bangladesh

**DOI:** 10.3329/jhpn.v31i1.14745

**Published:** 2013-03

**Authors:** Haribondhu Sarma, Elizabeth Oliveras

**Affiliations:** ^1^icddr,b, Mohakhali, Dhaka 1212, Bangladesh;; ^2^Pathfinder International, Nine Galen St., Suite 21, Watertown, MA 02472, USA

**Keywords:** Course curriculum, Health education, HIV and AIDS, Teachers training, Youth, Bangladesh

## Abstract

School-based HIV/AIDS education is a common and well-proven intervention strategy for providing information on HIV/AIDS to young people. However, lack of skills among teachers for imparting sensitive information to students can lead to programme failure in terms of achieving goals. A cross-sectional study was conducted among teachers to identify the factors that support or hinder their role in HIV/AIDS education. A self-administered questionnaire was used for interviewing teachers from randomly-selected schools in two adjacent districts in Bangladesh. Based on exposure to teachers’ training, the districts were divided into control and intervention areas and the teachers’ ability, skill, and their participation in HIV/AIDS education were compared between the districts. Trained teachers in the intervention schools were more likely to participate, less likely to face difficulties, and more likely to use interactive teaching methods in HIV/AIDS classes compared to the controls who did not receive any training. Inadequate allocation of time for conducting the HIV/AIDS class was found to be barriers to HIV/AIDS education that suggest the need to provide teachers with more support in terms of training and logistics.

## INTRODUCTION

Around the world, young people are at the centre of the HIV epidemic; almost half of all HIV-infected people are aged 15 −24 years (1). Southeast Asia and the Pacific region have the second highest prevalence of HIV with an estimated 1.27 million young people (in 2007) living with HIV (2). Many factors increase the vulnerability of young people to HIV but lack of knowledge has been identified as one of the leading factors (3,4). While many channels exist through which information can be provided to young people (5), interventions through the education sector have been implemented throughout the world to reach a large number of young people easily (6). Results of studies indicate that the school-based HIV/AIDS education programmes can result in significant changes in knowledge and attitudes that affect sexual behaviour of young people, leading to significant increases in the use of condom and reductions in sexual health problems, such as unwanted pregnancy, sexually transmitted infection, and abortion (6-10).

Effective school-based education on HIV/AIDS ideally encompasses two elements: curriculum development and training of teachers. Kirby *et al.* reviewed the evaluations of multiple studies of such interventions and found that successful curriculum-based programmes have 17 characteristics (6). Five of the 17 characteristics involve the development of the curriculum; eight involve the curriculum itself; and four describe the implementation of the curriculum, including selection and training of teachers with desired characteristics (6). Once a curriculum is developed, teachers need training to enable them to improve students’ knowledge about HIV prevention and transmission, attitudes toward HIV prevention, and behaviours relating to HIV/AIDS. Training and knowledge can ensure that they become a source of correct information and trusted persons with whom young people can raise sensitive and complicated issues about sexuality (11). This requires training on specific content which will enable the examination of their attitudes toward HIV prevention. It can also expose them to the participatory learning approaches that have been shown to ensure classroom discussion of HIV transmission and prevention (12). However, even when a well-designed HIV education programme is in place, some factors may affect its overall outcomes. Time allocated for a new HIV curriculum, availability of teachers, and distribution of teaching materials have all been shown to affect outcomes (11). Even in developed countries (13), factors, such as competition for curriculum time, shortness of lesson, low priority by senior personnel, limited experience of teachers, inability to use interactive teaching methods, and absence of teachers in school hinder sexual and HIV education programmes. However, some factors, including commitment of senior personnel, intensive teachers’ training (13), and selection of relevant teachers, such as science teachers, facilitate these programmes.

As part of the national HIV/AIDS prevention project in Bangladesh, PIACT, a non-profit, non-governmental organization, has introduced a curriculum for HIV/AIDS education for all secondary schools and higher secondary colleges of Grade 6 to 12. A separate teachers’ training was provided on this new curriculum. The curriculum includes age-appropriate information at each grade, given the sensitivity of the information. A cascading system was used in which master trainers were trained first to impart training to the core trainers who, in turn, trained subject teachers in schools. On an average, 3-5 subject teachers were trained from each school. All training focused on factual information about HIV and facilitative skills to teach the curriculum, including methods how to organize group discussions, puzzle games, role-plays, and how to encourage active participation of students. Training manuals, teachers’ guides, and supplementary materials were developed to meet the needs of teachers at different experience levels and to facilitate training.

This is the first time that an HIV/AIDS curriculum has been implemented in Bangladesh, and, to our knowledge, there is no study ever conducted in the country to understand the teachers’ role in HIV/AIDS prevention activities. Therefore, this study was designed to identify the factors that support or hinder the role of secondary school teachers in HIV/AIDS prevention activities in Bangladesh.

## MATERIALS AND METHODS

This study was cross-sectional in design; the teachers’ ability, skill, and their participation in HIV/AIDS education were compared between two adjacent districts—Mymensingh and Kishoreganj which were historically in a same bigger district of Dhaka division. The districts were selected purposively based on exposure to the HIV/AIDS training programme. A teachers’ training programme had been implemented in Mymensingh which was considered the intervention area but none in Kishoreganj which was treated as the control area. Geographic access was a key consideration in choosing these two districts. Both the districts can be reached from Dhaka within 2-3 hours. Culturally, both the districts are similar. However, Kishoreganj is more rural, and its population is more geographically scattered.

The study population included subject teachers in secondary schools responsible for teaching general and/or social sciences in grades 6-10 in classroom settings. These teachers were responsible for teaching the HIV/AIDS curriculum. We targeted secondary schools, including madrasas because students in this age-group are at a point in life when they are just beginning to explore their sexuality, and this may make teaching of the curriculum particularly challenging.

In each area, 90 schools were randomly selected from lists of schools provided by PIACT (the NGO conducting the training in the intervention area) and the local education department (in the control area). Schools were then selected using the simple random-sampling procedure in the SPSS software (version 11.5). We have calculated sample-size on a 2-sample comparison of proportions considering 95% confidence and 80% power. For calculation, we have used a conservative estimate of 50% prevalence in the control group and a 15% improvement in the intervention group. Given the estimation that the calculated sample-size is 333 per group and an estimated 4 teachers per school, 90 schools were considered adequate to obtain the needed sample. However, in the final analysis, we included 81 schools from the intervention area and 71 schools from the control area.

A pre-tested, self-administered questionnaire was used for collecting data during September-October 2008. The questionnaire included both open- and closed-ended questions. It addressed the level of knowledge on HIV/AIDS, attitudes (personal beliefs and values relating to HIV/AIDS messages included in the curriculum), and views on the value and appropriateness of teaching HIV/AIDS to school students and levels of comfort and confidence in teaching about HIVAIDS. Problems faced by the teachers and support offered to them by others were also assessed. Finally, questions about practical implementation in the classroom were asked, which included topics, such as the amount of available time for teaching about HIV/AIDS, adequacy of the time available, level of engagement by students during discussions on HIV/AIDS, type of students taught, and teaching methods followed. If new teaching methods introduced by the training were used, comfort in using those methods was also explored.

Trained field research assistants (FRAs) visited each school and sought permission from the headmaster, and written consent was obtained from the subject teachers to conduct the survey. The field team arranged questionnaire-administration session at each school. They briefed each teacher about the study objectives and procedure and obtained individual consent before copies of the questionnaire were distributed. All eligible teachers in a school, who were present on the day of the interview, were invited to participate. All the participating teachers of a school completed the questionnaire during the same session. The field team made the sitting arrangement in such a way that there was a reasonable distance between the participants to ensure confidentiality and to stop discussion among them. The FRAs observed the completion of the questionnaire, responded to the participant's questions regarding the questionnaire, and maintained field notes on the data-collection process.

We reviewed short-answer responses and developed code lists. We edited, compiled, and coded all data before entering these into the computer, using the Epi Info 6 software. Data were analyzed using the SPSS software (version 11.5) and R software (version 2.15.0). We ensured the quality of data through monitoring, supervision, and checking for the consistency of data. For performing cluster analysis, we have considered each school as a unit (containing a group or cluster of teachers). These schools were selected randomly, and we have drawn inference on these rather than individual teachers. Different results are obtained on schools both in the intervention and the control areas by accumulating results on individual teachers in the respective schools. The test statistical values and the p values of these tests are also given here. Due to not meeting the assumption of the number of successes and failures, some tests were not conducted.

## RESULTS

The participants included 353 teachers from 81 schools in the intervention area and 352 teachers from 71 schools in the control area. Once the desired sample-size was reached, no additional schools were enrolled. On average, 3-5 subject teachers were interviewed from each school. Overall, 96/801 (12%) teachers were not available at school on the day when the questionnaire was administered. This rate was higher in the intervention area compared to the control area because schools in the intervention area were reached during the Ramadan holidays.

Most (85%) teachers were from rural areas, with little variation between the intervention and the control district. The large majority of the teachers (77%) were enrolled in the study from co-education schools, reflecting the structure of the school system in Bangladesh. The mean age of the participants was 40 years. Nearly half of them in both the areas had at least either a bachelor's degree or a bachelor's degree with BEd (Bachelor of Education). The majority of the teachers (66%) in both the areas had been working in their current schools for 10 years or more (Table 1).

**T1able 1.** Characteristics of teachers and schools both in the intervention and the control district (%)

Characteristics	Intervention district (n=353)	Control district (n=352)	Total (N=705)
Location			
Rural	90	85*	85
Urban	10	15	15
Type of school			
General secondary school	75	80	78
Madrasa	25	20	22
Sex			
Male	87	76*	82
Female	13	24*	18
Age (years)			
22-29	9	19*	14
>29-39	39	32	35
>39-49	36	30	33
>49	17	19	18
Education (highest degree)			
HSC or below	15	15	15
Bachelor's degree	18	21	19
Bachelor's with BEd	51	48	49
Masters	5	7	6
Masters with BEd or MEd	12	9	11
Duration of teaching in current school (years)			
0-4	9.6	18.5	14
>4-9	21.0	19.9	20
>9-19	42.5	38.5	41
>19	26.9	23.3	25

*p<0.05; BEd=Bachelor of Education;

MEd=Master of Education

### Participation of teachers’ in HIV education

The subject teachers who received training (85%) were almost twice as likely to have taught about HIV in the classroom compared to those who did not receive the training (43%). The teachers were less likely to teach about HIV/AIDS to students in the lower grades. For example, although a teacher may have taught classes from Grade 6 to Grade 10 s/he may have taught about HIV/AIDS only to students of Grade 9. HIV was taught by more teachers per school in the intervention area than in the control area. In 51% of schools in the control area, none of the teachers taught about HIV; this was true in only 19% of the schools in the intervention area (Table 2). Most (73%) teachers who taught about HIV/AIDS taught boys and girls together, although about 20% taught girls alone. Of those who taught both boys and girls, in a few cases, they taught girls and boys separately; this was more common in the control area (9%).

**T1able 2.** Proportion of teachers who taught about HIV/AIDS in control and intervention schools

Percentage of teachers who taught about HIV	Percentage of schools in intervention district (n=81)	Percentage of schools in control district (n=71)
None	19	51
1-50	17	34
>50-75	33	6
>75-100	31	9

### Use of interactive teaching methods

When the teachers were asked what teaching methods they had used, most (91%) cited traditional teaching methods, such as lectures and question-and-answer sessions. In all schools of both the study areas, at least one teacher used traditional teaching methods. In the intervention district, at least one teacher in 97% (95% CI 93.85–100) schools used interactive teaching methods whereas, in the control district, at least one teacher used such methods in only 41% (95% CI 28.21-53.99) schools, and the use of interactive teaching methods has a significant difference (p<0.001) between the schools in two districts (Table 3).

The use of interactive training methods was positively associated with asking questions by students during lessons, particularly among the teachers who used group discussions and case study methods. In a separate analysis, about 91% of the teachers who used group discussion mentioned that their students asked questions compared to 77% of the teachers who did not use this method (p<0.001). The findings were similar in the case of the use of case study method.

### Facing difficulties while teaching about HIV/AIDS

Of the teachers in the intervention area, who taught about HIV/AIDS in the classroom, 13% did not complete the HIV/AIDS chapter. However, slight variations were observed between the teachers in general secondary schools and madrasas; 16% of madrasa teachers (n=91) did not complete the chapter compared to 12% of general secondary school teachers (n=359). Of those who did not complete the chapter, the most commonly-avoided content was sexual relationships, which 82% of the teachers in general secondary schools and 92% of the teachers in madrasas avoided. The main reason for avoiding such a chapter in the class was that students (70%) and teachers (26%) felt shy. The reason did not differ between the intervention and the control area.

When asked specifically whether it was easy or difficult to talk about HIV transmission and prevention, at the school-level analysis, there were about 57% (95% CI 46.05-68.15) schools in which at least one teacher faced difficulty while talking about HIV transmission in the intervention district, which is slightly larger (61%, 95% CI 47.91-73.49) in the control district. In about 49% (95% CI 38.23-60.57) schools in the intervention district, at least one teacher faced difficulty while talking about HIV prevention; the figure was much larger (70%, 95% CI 57.55-81.65) in the control district; facing such difficulties was significantly different (p=0.031) between the two groups of schools. In more schools, teachers reported that they faced difficulty while talking about HIV issues in the control district compared to the intervention district (Table 3).

### Benefit of supplementary materials

The subject teachers reported that the supplementary materials were effective in supporting teaching about HIV/AIDS in a classroom setting. Although it was assumed that all teachers in the intervention area received a teaching guide, only 61% had actually received it. Even among teachers who acted as core trainers and taught in an HIV class, 17% did not receive the guide. However, the teachers in the intervention area, who received the guide, found it easier to talk about HIV prevention than the teachers who did not receive it (71% vs 57%; p<0.01); they were more likely to report that they could create a comfortable environment for talking about sexual relationships in the classes (43% vs 31%; p<0.05).

**T1able 3.** Percentage of schools with 95% confidence intervals (CI) and results of proportion test in which teachers reported teaching about HIV/AIDS and participated in interactive teaching methods, faced difficulties, received community supports, and allocated time for imparting HIV/AIDS education in classroom setting

Item	Intervention (n=77)		Control (n=56)		Proportion test result for the two districts	
	% of school	95% CI	% of school	95% CI	Test statistical value	p value
Teaching methods						
Traditional methods	100	-	96.4	91.52-100	-	-
Interactive methods	97.4	93.85-100	41.1	28.21-53.99	50.191	<0.001
Difficulties faced						
Talking about HIV transmission	57.1	46.05-68.15	60.7	47.91-73.49	0.055	0.815
Talking about HIV prevention	49.4	38.23-60.57	69.6	57.55-81.65	4.676	0.031
Community support received from						
School Managing Committee	94.8	89.84-99.76	25	13.66-36.34	66.777	<0.001
Parents	72.7	62.75-82.65	17.9	7.86-27.94	36.883	<0.001
Other teachers	94.8	89.84-99.76	41.1	28.21-53.99	43.980	<0.001
Allocated mean time for HIV/AIDS class						
Less than 40 minutes	44.2	33.11-55.30	41.1	28.21-53.99	0.031	0.859
40 or more than 40 minutes	55.8	44.71-66.90	58.9	46.01-71.79	0.031	0.859

### Creation of a supportive environment for teaching about HIV/AIDS

To create an enabling environment for teaching about HIV/AIDS in the classroom setting, the teachers who received training were more likely to arrange meetings with the community members, other teachers, and parents than the teachers in the control area. In the intervention area, such meetings were held at almost two-thirds of the schools. Where the meetings were conducted, not all teachers were involved. In 20% of the schools, less than half of the teachers participated in the meetings.

At school-level analysis, receiving community supports from the members of school managing committee, parents, and other teachers are significantly higher (p<0.001) in the intervention schools compared to the control area. In the intervention district, teachers in almost 95% (95% CI 89.84-99.76) schools reported that they got support from the members of school managing committees about HIV/AIDS-related classes, which is true for only 25% (95% CI 13.66-36.34) schools in the control district. Similarly, in the intervention area, teachers in 73% (95% CI 62.75-82.65) schools got support from parents; and teachers in 95% (95% CI 89.84-99.76) schools got support from other teachers; in the control district, these percentages were only 18% (95% CI 7.86-27.94) schools for parents, and 41% (95% CI 28.21-53.99) schools for other teachers (Table 3).

### Time for HIV/AIDS class-session

The teachers felt that the time allocated for each of the HIV/AIDS classes was insufficient. Although the teachers had 40 minutes officially allocated for each class, in over half of the schools (56%, 95% CI 44.71-66.90) in the intervention area, at least one teacher reported having 40 minutes or more to conduct each HIV/AIDS class, which was almost similar (59%, 95% CI 46.01-71.79) in proportion of schools in the control area. In terms of mean allocated time for HIV/AIDS class, there was no statistical significance between the schools in these two districts (Table 3). When the teachers were asked whether the allocated time was adequate for conducting the HIV/AIDS class, about half of them mentioned that the allocated time was not enough.

## DISCUSSION

The results of the study suggest that simply introducing HIV/AIDS topics into the curriculum is not sufficient to ensure that it is implemented. In the control district where teachers did not receive training, less than half of them had taught HIV to their students. The teachers in the district where training was provided were more likely to have taught about HIV at all grade levels, particularly to the students of Grade 6 and 7. Exposure to training was also associated with lower levels of difficulties in teaching about HIV/AIDS at the classroom setting. No community members, including parents, actively opposed the teaching about HIV/AIDS in the classroom. The barriers to HIV/AIDS education in the classroom were quite practical; for example, the time for teaching was not adequate, and the teaching guide was not received by all the teachers.

Training of teachers on the new HIV/AIDS curriculum was positively associated with the development of their individual skills and building of confidence, and this finding directly correlated with the findings of other studies (14). Training on HIV/AIDS education increased the skills of teachers, which made them more capable to teach about HIV/AIDS-related issues and overcome the barriers, particularly talking to students about sexuality by using interactive teaching methods in the classroom setting. The interactive teaching method seems to be more effective in teaching about HIV/AIDS, and, on such methods more emphasis was given during training. There is a clear difference between the intervention and the control district in using such method by the teachers. In the intervention district, at least one teacher used such methods in significantly higher proportion of schools compared to the control schools, indicating the necessity of training to the teachers.

In more schools in the control district, higher number of teachers reported that they face difficulty while talking about HIV issues compared to the intervention district. This also demonstrated the lack of skills of the teachers and indicated the training need among them. Similar findings were observed in several studies of teachers feeling uncomfortable in discussing sexuality-related matters (15-18) and suggested that training of teachers should address this issue effectively (19).

One anticipated obstacle—opposition from the community—was not found to be a problem, except for a few teachers in some schools. No community members actively opposed the teaching about HIV/AIDS in the classroom. This finding contrasts with the findings of other studies in low- and middle-income countries where the community plays a strong role in advocating against sexual health education (20,21). In many schools, teachers reported that they did not receive any negative response from the community members, particularly from the local elites and parents. However, more active support might make teachers more comfortable in discussing these sensitive issues with their students (22). Meetings with the local elites and parents ensured more active support from them. In more schools in the intervention area, teachers were holding such meetings. Underlining such importance, the teachers should be encouraged in their training sessions to hold such meetings with the members of school managing committees, community stakeholders, and other teachers, alongside conducting the HIV classes.

This assessment also identified factors from the school level that held additional challenges to implementing the curriculum, and these challenges are consistent with findings in other settings (13-15, 21,23). For example, the teachers struggled with inadequate time for teaching about HIV/AIDS, and the time constraint did not allow some teachers to complete the HIV chapter and is a potential obstacle to the full implementation of the curriculum (21). Consideration should, thus, be given to providing both additional time for teaching and training in managing sessions within a 40-minute class.

Another barrier identified at the programme implementation level was inadequate distribution of teaching materials. About 40% of the teachers did not receive the materials, although the findings highlight the importance of the teaching guide provided to the teachers in the intervention area. Those teachers who received it were more able to discuss HIV prevention with their students and to create a comfortable atmosphere for discussing sexual relationships. Both factors are generally related to the success of school-based HIV-prevention programmes (4).

### Limitations

The study originally did not take into account the clustering when calculating the sample-size. As a result, there are some concerns whether the study would have adequate power to detect any difference between the intervention and the control groups. This study included only two districts for rapid assessment. The results obtained from the study in these two districts may not be generalizable throughout the country, particularly given that there is a greater religious diversity in these districts than in other areas and that these are not particularly conservative areas. We used a self-administered questionnaire for data collection, which is not a common technique used in Bangladesh. As a result, there are some concerns whether the respondents understood the questions. However, the respondents were teachers of secondary schools, and 85% of them had at least a bachelor's degree; so, it is likely that they were able to respond to the questions effectively as was demonstrated during field-testing. Moreover, administration was proctored by the field team, and any questions about the tool were answered and clarifications made.

### Conclusions

The findings of the study demonstrated that the implementation of the HIV/AIDS education programme in school settings was enhanced through training of teachers. This finding is reinforced by the fact that a higher percentage of teachers who received training reported active participation on the part of the students. Globally, there are a few examples that HIV/AIDS information has been provided to the young people through the formal education system. The findings give new thought in implementing HIV/AIDS education programmes in a low-epidemic country, like Bangladesh where HIV/AIDS is believed to be highly sensitive in terms of the sociocultural and religious contexts. However, even in the district where training was provided, less than half of the teachers used interactive methods, suggesting that more work is needed in this regard. Addressing a few key issues, such as making materials available and helping teachers to teach within the available time, may improve the implementation of the HIV/AIDS curriculum in future. Moreover, the results of the study indicate that teachers’ training programme needs to be improved, considering the practical barriers to implementing the HIV/AIDS curriculum successfully.

## ACKNOWLEDGEMENTS

The study was funded by the Ministry of Health and Family Welfare (MoHFW), Government of the People's Republic of Bangladesh and the Global Fund to Fight AIDS, Tuberculosis and Malaria (Grant No. BAN-39724). The authors acknowledge the commitment of Save the Children–USA, the management agency of the project to this research effort. The authors also thank all the subject teachers who participated in the study. The authors are grateful to Robin Morrison Martz for reviewing the earlier draft of the manuscript and Rashedul Haque for his support during data analysis. The authors also thank the Field Research Supervisors and Field Research Assistants who helped during data collection.
